# Mapping internal connectivity through human migration in malaria endemic countries

**DOI:** 10.1038/sdata.2016.66

**Published:** 2016-08-16

**Authors:** Alessandro Sorichetta, Tom J. Bird, Nick W. Ruktanonchai, Elisabeth zu Erbach-Schoenberg, Carla Pezzulo, Natalia Tejedor, Ian C. Waldock, Jason D. Sadler, Andres J. Garcia, Luigi Sedda, Andrew J. Tatem

**Affiliations:** 1 WorldPop, Geography and Environment, University of Southampton, Highfield Campus, Southampton SO17 1BJ, UK; 2 Flowminder Foundation, Roslagsgatan 17, Stockholm SE-11355, Sweden; 3 Institute for Life Sciences, University of Southampton, Highfield Campus, Southampton SO17 1BJ, UK; 4 GeoData, University of Southampton, Highfield Campus, Southampton SO17 1BJ, UK; 5 Bill and Melinda Gates Foundation, 440 5th Ave N., Seattle, Washington 98109, USA; 6 CHICAS, Lancaster Medical School, Lancaster University, Lancaster LA1 4YG, UK

**Keywords:** Malaria, Geography, Public health, Environmental chemistry

## Abstract

Human mobility continues to increase in terms of volumes and reach, producing growing global connectivity. This connectivity hampers efforts to eliminate infectious diseases such as malaria through reintroductions of pathogens, and thus accounting for it becomes important in designing global, continental, regional, and national strategies. Recent works have shown that census-derived migration data provides a good proxy for internal connectivity, in terms of relative strengths of movement between administrative units, across temporal scales. To support global malaria eradication strategy efforts, here we describe the construction of an open access archive of estimated internal migration flows in endemic countries built through pooling of census microdata. These connectivity datasets, described here along with the approaches and methods used to create and validate them, are available both through the WorldPop website and the WorldPop Dataverse Repository.

## Background & Summary

According to the International Organization for Migration^1^ and The World Bank^2^, without accounting for seasonal and temporary migrants, more than 1 billion people are currently living outside their places of origin, with about 740 million of them classified as internal migrants. Additionally, in 2014 around 67 million passengers travelled on international and domestic flights every week^3^ and hundreds of millions are estimated to commute daily by public transport and private vehicles^4^. Human mobility is expected to continue rising in volume and reach, producing increasing global connectivity that has a range of impacts, including rising numbers of invasive species, the spread of drug resistance, and disease pandemics. In this context, quantifying human mobility across multiple temporal and spatial scales, becomes crucial for quantifying its effects on society^5–7^, evaluating its relationship with the environment^8,9^, better understanding human-related processes such as urbanization and land use change^10–13^, and providing a strong evidence base to support both development^14–16^ and public health^17–19^ applications and policies.

In public health, the role of human mobility in the spread of infectious diseases is exemplified by the presence of HIV/AIDS in areas outside where it first emerged at the beginning of the twentieth century^20–22^, the 2003 SARS epidemic^23^, the 2007 Chikungunya outbreaks in Italy and France^24,25^, the 2009 H1N1 pandemic^26^, the 2014 Ebola outbreak in Western Africa^27^, the resurgence of malaria cases in areas where the disease was once eliminated^28^, and the worldwide spread of drug resistant pathogens^29^. Consequently, it is clear that to provide better informed guidelines, both at the national and international level, the effects of human mobility and connectivity in driving disease dynamics need to be better understood and accounted for refs 30–33.

Local malaria elimination and global malaria eradication are rising up the international agenda^34–36^. Evidence from the previous global malaria eradication program^37^, as well as from recent studies, control campaigns, and elimination efforts^38–41^ highlight the importance of accounting for human mobility in designing elimination plans. Infected people may unknowingly transport malaria parasites (potentially including antimalarial-resistant strains^42^) into new areas. Parasites can be imported either from other countries^43^ or from other areas within the same country^44^. Thus, because of the flow of imported cases from high- to low-transmission settings, the latter will face difficulties in achieving elimination and maintaining malaria-free status if it is achieved^43^. Nevertheless, despite the importance of these dynamics being long recognized^45,46^, attempts to translate human mobility model outputs into malaria policy are still rare^47^.

As detailed in Tatem^7^, sources of human mobility data potentially useful for modelling pathogen movements include: air and sea travel data records (including open access modelled versions of them); census migration data; travel history and displacement surveys; GPS tracking data and volunteered geographic information (with the latter including geolocated social media data), and even satellite night-time light data. In particular, patient travel history data, containing detailed demographic information and travel motivations, are traditionally used to understand malaria parasite importation patterns^48–50^. Recently, mobile phone call detail records (CDRs) have been increasingly used for measuring short-term human movements^51,52^ and thus, either alone^38,53,54^ or in combination with travel history data^55^ and malaria case data, for supporting malaria control and elimination strategic planning.

However, because of difficulties in sharing and accessing CDRs (mostly due to commercial and privacy concerns)^7,56,57^, alternative datasets are required in order to quantify and map internal connectivity across continental scales. To this end, using CDRs, Wesolowski *et al.*^56^ and Ruktanonchai *et al.*^58^ demonstrated that widely-available and easy-to-obtain census-derived internal migration flow data can serve as reliable proxies for the relative strength of within-country human connectivity across multiple temporal scales.

**Figure 1:**
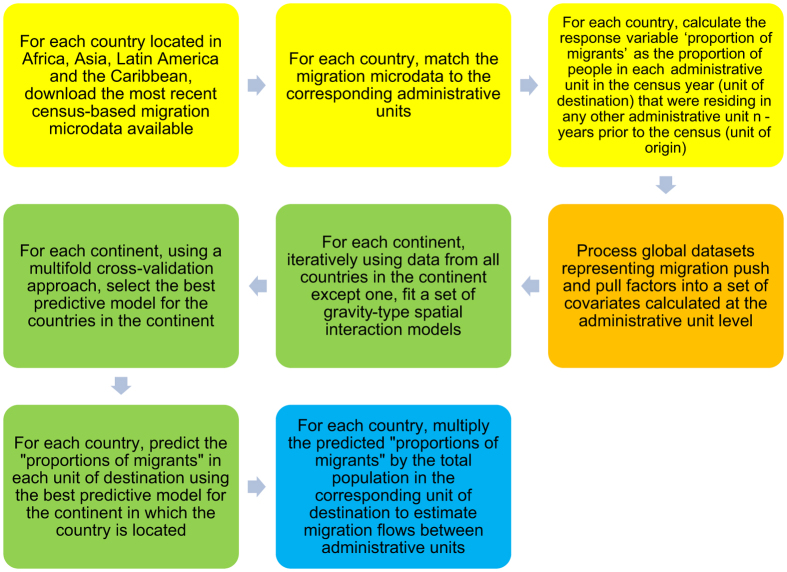
Schematic overview of the approach used to estimate the 5-year (2005–2010) internal human migration flows for every Pf and Pv endemic country. The preparation of the response variable and covariates is described in the yellow and orange panels, respectively. The modelling steps are outlined in the green panels and the estimation of the 5-year internal migration flows is described in the blue panel.

Within the framework of the WorldPop Project (www.worldpop.org), and following the approaches described in Henry *et al.*^59^ and Garcia *et al.*^60^ (Fig. 1), internal census-based migration microdata available through the online IPUMS-International (IPUMSI) database^61^, along with a number of other ancillary datasets, were assembled and processed to produce an open access archive of estimated 5-year (2005–2010) internal human migration flows for every *Plasmodium falciparum* and *Plasmodium vivax* (hereafter simply referred as *Pf* and *Pv*, respectively) endemic country^62,63^ (Supplementary Table 1).

## Methods

### Estimating internal migration flows between administrative units

Following Garcia *et al.*^60^ a gravity model-based approach was used to estimate the total number of people migrating from one administrative unit to any other administrative unit, between 2005 and 2010, within each malaria endemic country located in Africa, Asia, Latin America and the Caribbean^62,63^ (Supplementary Table 1).

The simplest gravity-type spatial interaction model, proposed by Zipf^64^, considers the total population in a location of origin *i* and in a location of destination *j* (henceforth simply indicated as *i* and *j*), and the distance between the two locations to predict the migration flow (*MIG*_*ij*_) between them. Thus, migration flows between administrative units can be estimated using the following function:

(1)MIGij=PiαPjβdijγ

where $P_{i}^{\alpha }$ and $P_{j}^{\beta }$ represent the populations in the location of origin *i* and of destination *j*, respectively, and $d_{\mathrm{ij}}^{\gamma }$ represents the distance between *i* and *j*; with *α*, *β*, and *γ* being parameters, used to indicate the magnitude of the effect for each covariate, that are typically estimated in the statistical modelling framework.

In this study, following the notation from Henry *et al.*^59^ and Garcia *et al.*^60^, the basic gravity-type spatial interaction in equation (1) was extended in order to include additional geographical and socioeconomic factors described in detail in the Data collection and preparation subsection below. Since the census-based migration microdata extracted from the IPUMSI database^61^ represent only a sample of the total census, a logistic regression was used to model the proportion of people migrating between administrative units^65^. In particular, the logistic regression was used to model the proportion of people residing in *j* in the census year who were in *i* ‘n’ years prior to the census. Thus, the proportion of migrants in *j* in the census year that were previously residing in *i* was estimated using the following logistic regression function:

(2)pij=eβ0+β1Pi+β2Pj−β3dij1+eβ0+β1Pi+β2Pj−β3dij

where $p_{ij}=MIG_{ij}/TOT_{j}$; with *MIG*_*ij*_ and *TOT*_*j*_ representing the number of people residing in *j* in the census year that were in *i* ‘n’ years prior to the census and the total population residing in *j* in the census year, respectively.

Initially, a separate vector **β**=(β_0_, β_1_, …, β_n_) of coefficients was used in the linear predictor of the gravity model for each country (including malaria non-endemic countries), in Africa, Asia, Latin America and the Caribbean, for which migration data were available in the IPUMSI database^61^ (hereafter referred as IPUMSI countries; Table 1).

However, since the main aim of this study was to estimate internal human migration flows for malaria endemic countries for which migration data are not available, ultimately, models where the linear predictors were common across all countries located in the same continent were constructed (under the assumption of homogeneity of the process along the space). To investigate possible nonlinear relationships, models where linear predictors were replaced by additive predictors, using a Generalized Additive Modelling (GAM) framework^66^, were also explored.

**Figure 2:**
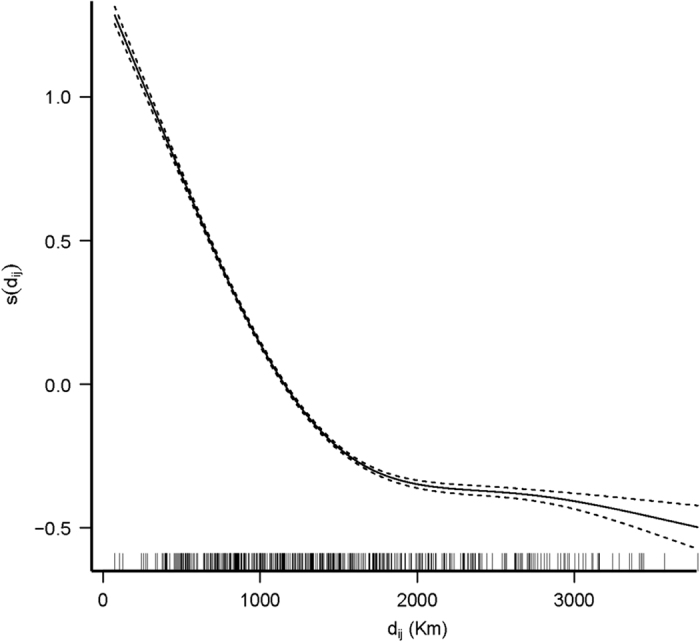
Variation of the effect of the distance between administrative units (dij) on the predicted proportion of migrants in j in the census year that were previously residing in i (solid line) and 0.95 confidence intervals (dashed lines) as estimated by using a GAM. The rug plot (i.e., the vertical lines along the x axis) represents the distribution of the observed dij values. This example shows the result obtained using data for all countries located in Latin America and the Caribbean.

GAM is a type of regression that, while preserving the functionality of using linear terms, allows covariates to have different and possibly opposite effects on the response variable by incorporating regression coefficients with smooth nonlinear form (Fig. 2).

Thus, all possible combinations of covariates (listed in Table 2 and Supplementary Table 2) were tested in a logistic regression model and then only the linear predictors of all continuous covariates of the best predictive logistic regression model were also modelled using a GAM.

**Figure 3:**
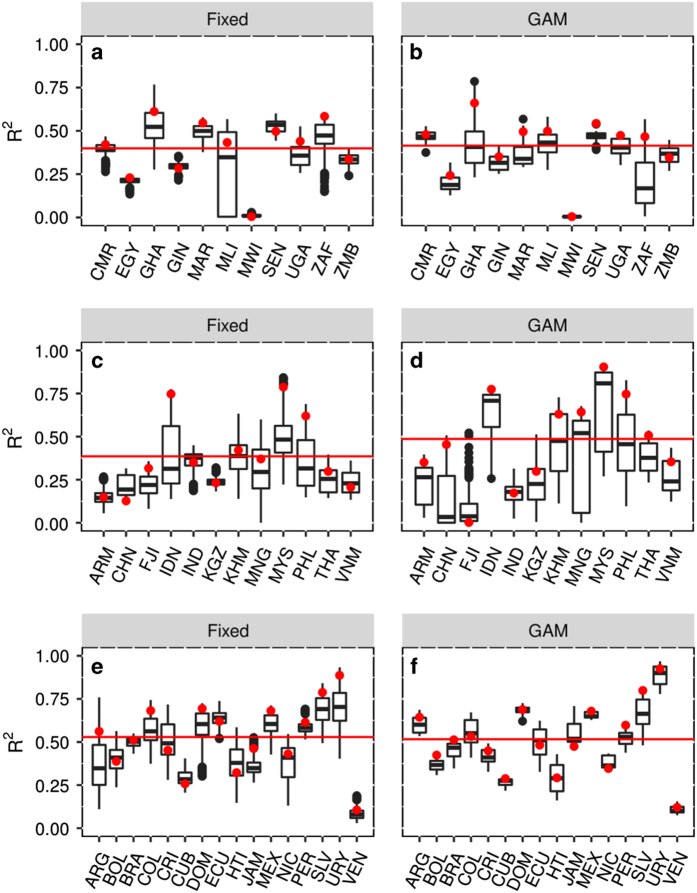
Boxplots showing the distribution of all R2-values, for each withheld country, for all logistic regression (**a**,**c**,**e**) and GAM (**b**,**d**,**f**) models explored for Africa (**a**,**b**), Asia (**c**,**d**) and Latin America and the Caribbean (**e**,**f**). The red lines represent the best averaged R2 values used to select the best predictive model for each continent (Supplementary Table 3) while the red dots represent the R2 values, for all withheld countries, calculated using the best predictive model referring to the continent in which they are located.

For each continent, the overall combinations of covariates and model types were explored using a multi-step approach to identify the model with the greatest predictive power in countries for which migration data were not available. The best model was then selected using a leave-one-out cross-validation approach^67^ in which the observed proportion of migrants in *j* previously residing in *i* for all countries except one were used for fitting models, that were subsequently used to predict the proportion of migrants in *j* previously residing in *i* in the withheld country. The correlation coefficient (R^2^) was selected to measure the variance explained after verifying homoscedasticity and testing overdispersion using a chi-squared test. This process was then repeated through iteratively withholding one country at the time. For each model, the R^2^ values for all withheld countries were averaged and used to rank each models according to their predictive power averaged across all withheld countries (Fig. 3). The overall best predictive model for each continent (Supplementary Table 3) was then used to predict the proportion of migrants residing in *j* who were previously residing in *i* for every malaria endemic country located in the corresponding continent (refer to Supplementary Table 4a,b and c for summary statistics of each best predictive model for Africa, Asia, and Latin America and the Caribbean, respectively).

**Figure 4:**
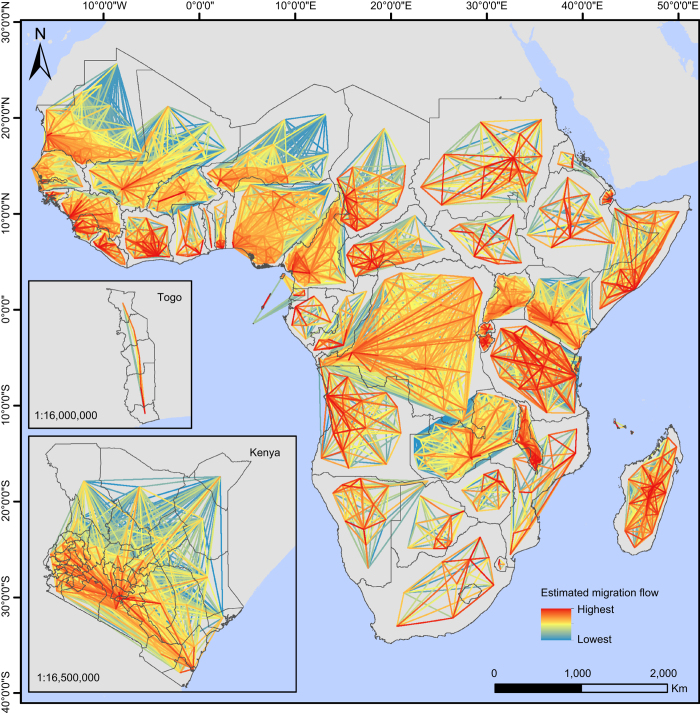
Estimated internal human migration flows between subnational administrative units for every malaria endemic country in Africa (Supplementary Table 1). Coordinates for all three panels refer to GCS WGS 1984. For illustrative purposes, subnational unit boundaries are shown only in the insets and the colour ranges used to represent the flows are country-specific (refer to Supplementary Fig. 1 for additional close-up views of internal migration flows in Africa).

**Figure 5:**
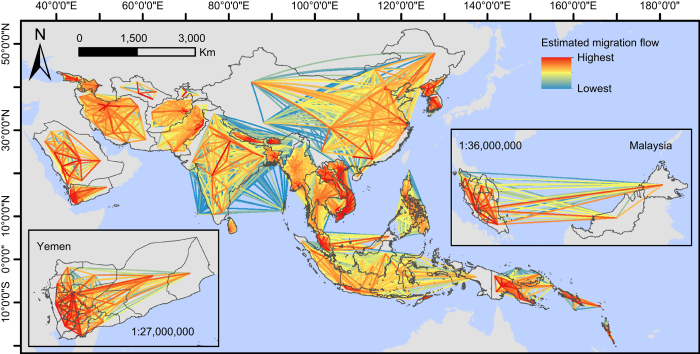
Estimated internal human migration flows between subnational administrative units for every malaria endemic country in Asia (Supplementary Table 1). Coordinates for all three panels refer to GCS WGS 1984. For illustrative purposes, subnational unit boundaries are shown only in the insets and the colour ranges used to represent the flows are country-specific (refer to Supplementary Fig. 2a,b for additional close-up views of internal migration flows in Asia).

**Figure 6:**
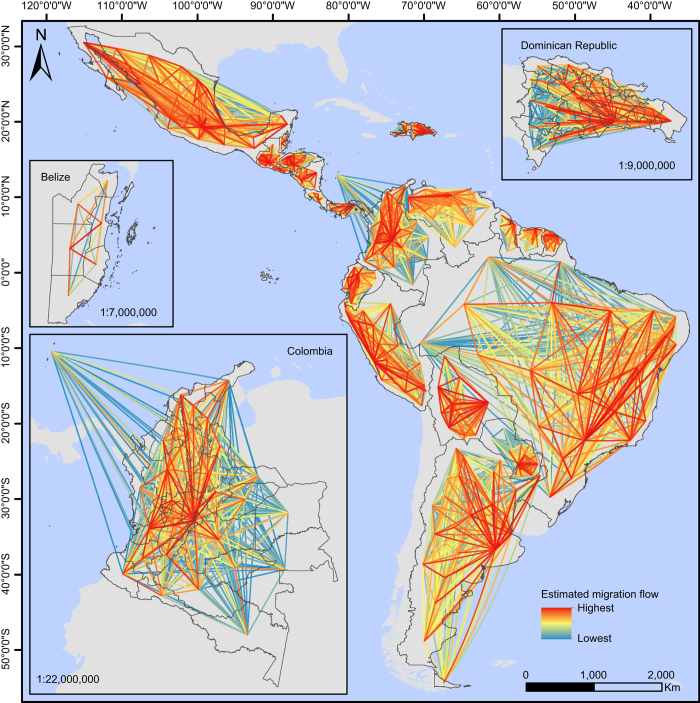
Estimated internal human migration flows between subnational administrative units for every malaria endemic country in Latin America and the Caribbean (Supplementary Table 1). Coordinates for all three panels refer to GCS WGS 1984. For illustrative purposes, subnational unit boundaries are shown only in the insets and the colour ranges used to represent the flows are country-specific (refer to Supplementary Fig. 3 for additional close-up views of internal migration flows in Latin America and Caribbean).

Finally, in order to estimate the total number of people that migrated from *i* to *j* between 2005 and 2010 (Figs 4,5,6), for each country the predicted proportion of migrants residing in *j* was multiplied by the 2010 total population in *j;* with the latter calculated using either the corresponding WorldPop^68–70^ or the Gridded Population of the World version 4 (GPWv4)^71^ dataset adjusted to match United Nations Population Division (UNPD) estimates for 2010 (ref. 72). Refer to the Data collection and preparation subsection section below for a detailed description of how the population datasets mentioned above were identified and used.

Both model selection and prediction were performed using an R^73^ script contained in the WorldPop-InternalMigration-v1 code^74^ briefly described in the Code availability subsection below.

### Data collection and preparation

In most of the countries available through the online IPUMSI database, internal migration variables were recorded by asking respondents either their administrative unit of residence 15, 5, or 1 prior to the census, or their previous residence and the number of years they are residing in the current locality. Considering that 5-year was the temporal interval available for most of the countries in the IPUMSI database and the fact that it has been demonstrated that both 1- and 5-year census-based internal migration data generally align well with shorter-term population movements in terms of relative strength of connections^56,58^, the 5-year migration data were used in this study. This maximised the amount of data that could be used to fit the gravity models subsequently used for predicting internal migration flows for every malaria endemic country. Thus, for each country listed in Table 1, harmonized, census-based 5-year internal migration data were extracted from the most recent census microdata available through the IPUMSI database^61^, downloaded locally, and eventually uploaded into a PostgreSQL database using a Microsoft Visual Studio 2010 user interface. The IPUMSI data stored in the PostgreSQL database were subsequently queried, using SQL, to quantify the number of people that migrated from each subnational administrative unit *i* to every other subnational administrative unit *j* during the 5-year timespan. These numbers were then matched to the corresponding country administrative unit spatial dataset, extracted from either the Global Administrative Areas (GADM)^75^ or the Global Administrative Unit Layers (GAUL)^76^ database, in a GIS environment. This was done by manually adding a unique ‘ID’ to each spatial unit corresponding to the one in the PostgreSQL database (hereafter referred as ‘IPUMSID’). In some cases, depending on the country, either the spatial detail of the IPUMSI migration data had to be reduced to match the lower spatial detail of the corresponding administrative unit dataset or spatially contiguous units in the administrative unit dataset had to be merged together to match the lower spatial detail of the IPUMSI migration data. In some other cases, ‘IPUMSIDs’ had to be edited or spatially contiguous units in the administrative unit dataset had to be merged together to match the reorganisation of the administrative units during the 5 years prior to the census. Finally, before calculating the migration flows between administrative units, another SQL query was used to classify each person in the census sample as either an internal migrant (1) or not (0). Examples of SQL queries used to perform the tasks described above are included in the WorldPop-InternalMigration-v1 code^74^ briefly described in the Code availability subsection below.

### Response variable and covariates

For each country, the response variable, or the proportion of migrants residing in *j* in the census year that were residing in *i* 5 years prior to the census, was obtained by dividing the number of migrants residing in *j* in the census year that were residing in *i* 5 years prior the census by the total population residing in *j* in the census year; with both numbers based only on the information contained in IPUMSI census samples.

The administrative units spatially matching the IPUMSI migration microdata were used to calculate the distance between each pair of administrative units, their area, total population, and proportion of urban population. These main covariates (Table 2), along with other covariates derived from them (Supplementary Table 2), represent the pull and push migration factors, known to influence internal migration^59,60,77^, that were used to extend the basic gravity model proposed by Zipf^64^.

Other factors, including environmental factors^59,60^, and country-specific factors, such as literacy and percentage of male population^59^ or infrastructure and transportation^78^, were not used because (i) the factors listed in the previous paragraph alone proved to be able to explain most of the variance in the gravity models of Garcia *et al.*^59^, and (ii) only globally available datasets were explored in order to consistently model internal migration across all countries.

### Calculating response variable and covariates

For each country, the total population in each administrative unit was calculated using the corresponding WorldPop^79^ (Data Citation 1) or GPWv4 (ref. 80) population count raster dataset adjusted to match UNPD estimates for 2010^72^. The GPWv4 datasets were resampled to the spatial resolution of the WorlpPop datasets and used only for countries for which the WorldPop datasets were not available (Supplementary Table 1).

The area of each unit was calculated using each country vector administrative unit dataset projected to the most appropriate country-specific projected coordinate system, in order to minimize areal distortion, and ultimately reprojected to GCS WGS84.

The proportion of people in urbanized areas in each unit was calculated using the MODIS 500 m Global Urban Extent raster dataset^81,82^. The latter was converted to vector polygons, using the ArcGIS ‘Raster to Polygon’ tool^83^, and intersected with the reprojected country vector administrative unit dataset using the ArcGIS ‘Intersect’ tool^83^. Then, both the intersect output (containing polygons representing the total urban area within each unit uniquely identified by its ‘IPUMSID’) and the country vector administrative unit dataset were rasterized, at the resolution of the corresponding raster population dataset (i.e., 3 arc seconds 3 arc equals to approximately 100 m at the equator), and co-registered with it.

The two raster outputs, along with the population count raster dataset, were then input to the ArcGIS ‘Zonal Statistics as Table’ tool^83^ to generate two tables containing the total population and urban population in each unit (with the rasterized administrative units and thus their ‘IPUMSIDs’ used to define the zones). Subsequently, both tables were joined to the attribute table of the vector administrative unit dataset, using the ‘IPUMSID’ field to perform the join operation, and the proportion of urban population in each unit was calculated simply dividing its urban population by its total population.

The geodesic distance between each pair of administrative units, with the latter represented by their centroids, was calculated using the ArcGIS ‘Generate Near Table (Analysis)’ tool^83^. The ‘IN_FID’ and ‘NEAR_FID’ fields (identifying the administrative unit of origin and destination, respectively) in the output ‘distance’ table were then used for joining twice the ‘centroid attribute’ table using the centroid ‘ID’ field to perform the join operation. Since the ‘centroid attribute’ table contains the attributes of each administrative unit represented by the corresponding centroid, the join operation allowed to generate a ‘distance’ table containing all pairs of origin and destination administrative units along with their ‘IPUMSIDs’ and attributes including the unit’s area, total population, and proportion of urban population. Origin and destination ‘IPUMSID’ fields were then renamed ‘NODEI’ and ‘NODEJ’, respectively.

A ‘contiguity’ table containing information about spatial contiguity of administrative units (defined based on polygons sharing an edge) was generated using the ArcGIS ‘Generate Spatial Weights Matrix’ tool^83^ and subsequently joined with the ‘distance’ table to obtain a new table containing all main covariates, listed in Table 2, calculated at the unit level. This join operation (based on both the ‘NODEI’ and ‘NODEJ’ field the in the ‘distance’ table and the corresponding ‘IPUMSID’ and ‘NID’ field in the ‘contiguity’ table) was performed through two different R scripts depending on whether the country is an IPUMSI or a non-IPUMSI countries. In particular, the R script for the IPUMSI countries added to the new table a ‘MIGIJ’ field containing the number of people that migrated from each ‘NODEI’ to each other ‘NODEJ’ according to the IPUMSI migration microdata and calculated the response variable.

Finally, on a continent basis, all IPUMSI country tables were merged together and input to an R^73^ script that generated the additional covariates listed in Supplementary Table 2, identified the best predictive model for each continent, as described in the previous section, and was used to estimate the 5-year (2005–2010) internal human migration flows for every malaria endemic country using the best predictive model selected for the corresponding continent.

All operations described above, excluding the reprojection of the vector administrative unit datasets and the calculation of their surface areas, for all IPUMSI and non-IPUMSI countries, were performed using the WorldPop-InternalMigration-v1 code^74^ briefly described in the Code availability subsection below.

### Code availability

The WorldPop-InternalMigration-v1 code^74^, used to produce the open access archive of estimated 5-year (2005–2010) internal human migration flows described in this article, is publicly available through Figshare. It consists of 1) a Microsoft Visual Studio 2010 user interface allowing users to upload the IPUMSI census microdata to a PostgreSQL database; 2) example SQL queries that were used to match the spatial detail of the IPUMSI migration data to spatial detail of the corresponding administrative unit dataset and to identify internal migrants within the IPUMSI census samples 3) an ArcToolbox geoprocessing tool^82^ that assigns a unique ID to each administrative unit and calculates the corresponding total population and proportion of urban population; 4) a Python^84^/ArcPy^83^ script that creates two tables, one containing spatial contiguity information between each pair of administrative units (‘contiguity.csv’) and another one containing the ISO country code, the continent in which the country is located, the distance between each pair of administrative units, their total population, proportion of urban population, surface area, and the geographic coordinates (GCS WGS84) of their centroid (‘distance.csv’); 5) two R^73^ scripts, one for the IPUMSI countries used to query the IPUMSI migration microdata loaded in the PostgreSQL database, calculate the response variable, and join the query result with the two output tables of the python script, and another one for the non-IPUMSI countries used just to join together the two output tables of the python script; and 6) an R^73^ script that performs the model selection and estimates the 5-year (2005–2010) internal human migration flows between subnational administrative units.

All available sets of code are named progressively and must be run sequentially according to the order in which they are presented above. They are also internally documented in order to both briefly explain their purpose and, when required, guide the user through their customization.

## Data Records

All datasets described in this article, referring to all *Pf* and *Pv* endemic countries listed in Supplementary Table 1, are publicly and freely available both through the WorldPop Dataverse Repository (Data Citation 2) and the WorldPop website (http://www.worldpop.org.uk/data/data_sources/). However, it is important to note that while the datasets stored in the Dataverse Repository represent the datasets produced at the time of writing, and will be preserved in their published form, the datasets stored on the WorldPop website may be updated as more recent IPUMSI migration data for the countries listed in Table 1, become available. Similarly, the datasets stored on the WorldPop website may be updated as IPUMSI census-based migration microdata become available for additional malaria endemic and non-endemic countries located in Africa, Asia, Latin America and the Caribbean. Indeed, the availability of migration data for additional countries may enable further improvements of the predictive power of the gravity models used to estimate the internal migration flows. For each county, the corresponding internal migration dataset, along with a point dataset showing the nodes of the migration network, (Table 3) can be obtained by downloading the corresponding zipped archive associated with the continent in which the country of interest is located.

## Technical Validation

### Goodness of fit and error p-value

All countries available in the IPUMSI database were used to assess the accuracy of the predicted proportion of migrants in *j* in the census year that were previously residing in *i* 5 years prior to the census by comparing them with the corresponding observed values from the IPUMSI migration microdata. For each country, the goodness of fit (R^2^) between predicted and observed values and the corresponding error *P*-value, representing the average probability that predicted migration values lay outside the distribution of the observed values, are reported in Table 4 below. Both metrics were derived using (i) the observed IPUMSI migration flows from each *i* to any other *j* and (ii) the predicted IPUMSI-based migration flows calculated by multiplying the predicted proportion of migrants residing in *j* in the census year by the IPUMSI-based total number of people residing in *j* in the census year.

## Usage Notes

The estimated internal human migration flows between subnational administrative units can be used to support a range of applications from planning interventions, to measuring progress, designing strategies, and predicting response variables that are intrinsically dependent on migration flows and internal connectivity.

Ongoing work involves the integration of these datasets with malaria prevalence raster datasets^85–87^ in order to inform local elimination and global eradication planning by identifying subnational communities of malaria movement and sources and sinks of transmission within them^36,43,58^. Similarly, these datasets could be used to better model the spread and improve understanding of the drivers of the distributions of other infectious diseases, such as West Nile Virus, schistosomiasis, river blindness, and yellow fever, which are endemic in some of the countries listed in Supplementary Table 1. Additionally there are many uses of these data beyond infectious disease dynamics, in the fields of trade, demography, transportation and economics, for example.

There are a number of limitations, caveats, and assumptions inherent in the approach that should be considered when using the datasets outlined here. For consistency, internal migration flows were estimated using a fixed set of pull and push factors common to all countries and thus only a limited number of covariates were used to fit the gravity-type spatial interaction models and to create predictions. For this reason, as is a trade-off in the production of generalizable models, the model fit varied between countries and for some of them, such as Malawi, China, Cambodia, India, and Venezuela (Table 4), poor fits could be improved by considering additional, locally-specific migration drivers that could help to increase the percentage of variance explained^60,78^. Other limitations are the fact that migration models were fitted using only a small sample (ranging between 0.07 and 10%) of the full census for each country, and that in each sample a small number of households were swapped across administrative units. Moreover, the spatial detail at which migration is captured and summarized varies by country. Because of this, for some countries, the modelled role of some of the pull and push factors, may not have been captured at the spatial level at which they influence migration as recorded in the census. It is also important to consider that the underlying migration data are based only on permanent movements captured by the census and other types of migrations, such as seasonal movements and forced displacements, may be not captured by the model^88–90^.

The two main assumptions behind the approach presented here are that for each country (i) the census samples are considered to be representative at the administrative unit level at which migration was recorded and (ii) the percentage of people migrating between administrative units is considered to be constant over time. Regarding the second assumption, it is important to highlight that the use of census data from many years ago for some countries may have generated inaccurate estimates for the period considered in this study (i.e., 2005–2010), for example because of major changes in the countries’ socio-economic conditions from the time period covered by the census (e.g., the rapid economic development and urbanization that has occurred in China during the last two decades^91,92^). Similarly, in some other countries, either the presence of conflicts^93^ or the occurrence of natural disasters^88,89^ during the specific time period covered by the census may have produced fluctuations in the number of internal migrants and consequently biased results for the period considered in this study.

Finally, the estimated internal flows represent modelling outputs generated using ancillary covariate datasets, and thus, to avoid circularity they should not be used to make predictions or explore relationships with any of these ancillary datasets. It is also important to note that these ancillary datasets are modelling outputs in themselves and thus they have a degree of uncertainty that will carry over into the migration estimates.

## Additional Information

**How to cite this article:** Sorichetta, A. *et al.* Mapping internal connectivity through human migration in malaria endemic countries. *Sci. Data* 3:160066 doi: 10.1038/sdata.2016.66 (2016).

## Supplementary Material

Supplementary Tables

Supplementary Figures

## Figures and Tables

**Table 1:** Summary information about the edited IPUMSI 5-year internal migration microdata and the administrative unit datasets used to estimate the 5-year (2005–2010) internal human migration flows for every malaria endemic country

**Continent**	**ISO code**	**Census Year**	**Census sample (%)**	**No. of units**	**Unit level**	**Census data source**	**Administrative unit data source**
AFRICA	CMR	2005	10	58	2	Central Bureau of Census and Population Studies	GADM
AFRICA	GHA	2000	10	10	1	Ghana Statistical Services	GADM
AFRICA	GIN	1996	10	34	2	National Statistics Directorate	GADM
AFRICA	MWI	2008	10	31	1	National Statistical Office	GADM
AFRICA	MLI	1998	10	47	2	National Directorate of Statistics and Informatics	GADM
AFRICA	SEN	2002	10	34	2	National Agency of Statistics and Demography	GADM
AFRICA	ZAF	2007	2	9	1	Statistics South Africa	GADM
AFRICA	UGA	2002	10	56	1	Bureau of Statistics	GADM
AFRICA	ZMB	2010	10	72	2	Central Statistics Office	GADM
AFRICA	EGY	2006	10	27	1	Central Agency for Public Mobilization and Statistics	GADM
AFRICA	MAR	2004	5	18	1	Department of Statistics	GADM
ASIA	ARM	2001	10	11	1	National Statistical Service	GADM
ASIA	KGZ	1999	10	39	2	National Statistical Committee	GAUL
ASIA	IND	1999	0.07	32	1	Ministry of Statistics and Programme Implementation	GADM
ASIA	IDN	2010	10	27	1	BPS Statistics Indonesia	GADM
ASIA	THA	2000	1	76	1	National Statistical Office	GADM
ASIA	KHM	2008	10	24	1	National Institute of Statistics	GADM
ASIA	CHN	1990	1	30	1	National Bureau of Statistics	GADM
ASIA	MYS	2000	2	15	1	Department of Statistics	GAUL
ASIA	PHL	2000	10	77	1	National Statistics Office	GADM
ASIA	VNM	2009	15	63	2	General Statistics Office	GADM
ASIA	MNG	2000	10	21	1	National Statistical Office	GADM
ASIA	FIJ	2007	10	8	2	Bureau of Statistics	GADM
LAC	ARG	2001	10	24	1	National Institute of Statistics and Censuses	GADM
LAC	BOL	2001	10	35	1	National Institute of Statistics	GAUL
LAC	BRA	2010	5	27	1	Institute of Geography and Statistics	GADM
LAC	COL	2005	10	35	1	National Administrative Department of Statistics	GADM
LAC	CRI	2000	10	7	1	National Institute of Statistics and Censuses	GADM
LAC	DOM	2010	10	32	1	National Statistics Office	GADM
LAC	ECU	2010	10	23	1	National Institute of Statistics and Censuses	GADM
LAC	SLV	2007	10	14	1	Department of Statistics and Censuses	GADM
LAC	HTI	2003	10	10	1	Institute of Statistics and Informatics	GADM
LAC	MEX	2010	10	32	1	National Institute of Statistics, Geography, and Informatics	GADM
LAC	NIC	2005	10	15	1	National Institute of Information Development	GADM
LAC	PER	2007	10	25	1	National Institute of Statistics and Informatics	GADM
LAC	VEN	2001	10	23	1	National Institute of Statistics	GADM
LAC	CUB	2002	10	15	1	Office of National Statistics	GADM
LAC	JAM	2001	10	14	1	Statistical Institute	GADM
LAC	URY	2011	10	19	1	National Institute of Statistics	GADM
In the 1st column, LAC stands for Latin America and the Caribbean. In the 2nd column, countries are indicated using their ISO three letter country codes^94^ (refer to http://www.nationsonline.org/oneworld/country_code_list.htm for a list of all world countries and their ISO codes).							

**Table 2:** Summary information about the source datasets and the main covariates tested in the spatial gravity models and used to derive additional covariates (Supplementary Table 2) for improving the predictive power of the models.

**Dataset**	**Temporal coverage**	**Format**	**Type**	**Resolution**	**Source**	**Main covariate**
Subnational administrative unit boundaries	—	Vector	Categorical	—	GADM^75^	Distance (DISTIJ) and contiguity (CONTIJ) between administrative units and their area (AREAI and AREAJ)
Subnational administrative unit boundaries	—	Vector	Categorical	—	GAUL^76^	Distance (DISTIJ) and contiguity (CONTIJ) between administrative units and their area (AREAI and AREAJ)
Population count (adjusted to match UNPD estimates)	2010	Raster	Continuous	3 arc seconds	WorldPop^79^ Data Citation 1	Total population (POPI and POPJ) in each administrative unit
Population count (adjusted to match UNPD estimates)	2010	Raster	Continuous	30 arc seconds	GPWv4^80^	Total population (POPI and POPJ) in each administrative unit
MODIS 500 m Global Urban Extent	2000/2001	Raster	Categorical (binary)	15 arc seconds	Schneider *et al.*^81^	Proportion of urban population (URBANPROPI and URBAN PROPJ) in each administrative unit

**Table 3:** Name (ISO represent the country the dataset refers to), description, and format of all files available for each county listed in Supplementary Table 1

**Name**	**Description**	**Format**
ISO_5yr_InternalMigFlows_2010	Estimated 5-year (2005–2010) internal human migration flows between subnational administrative units.	CSV
ISO_AdminUnit_Centroids	Centroids representing the subnational administrative units used to estimate the 5-year internal human migration flows (with centroid ‘IPUMSIDs’ matching polygon NODEIs and NODEJs in the corresponding ISO_5yr_InternalMigFlows_2010.csv dataset).	SHP
ISO_AdminUnit_Edits_README	Description of the edits needed to match the spatial detail of the GADM/GAUL subnational administrative units to the spatial detail of the IPUMSI census-based migration microdata.	TXT
Readme files are distributed along with the other two datasets only if the administrative unit dataset has been edited, to match the spatial resolution of the IPUMSI migration microdata, before extracting the centroids.		

**Table 4:** Prediction accuracy of the best predictive models listed in Supplementary Table 3.

**Continent**	**ISO code**	**R**^**2**^	**Error** ***P*****-value**
AFRICA	CMR	0.60	0.07
AFRICA	EGY	0.21	0.20
AFRICA	GHA	0.68	0.21
AFRICA	GIN	0.39	0.09
AFRICA	MAR	0.52	0.14
AFRICA	MLI	0.51	0.14
AFRICA	MWI	0.02	0.06
AFRICA	SEN	0.54	0.12
AFRICA	UGA	0.50	0.11
AFRICA	ZAF	0.49	0.23
AFRICA	ZMB	0.37	0.22
ASIA	ARM	0.11	0.16
ASIA	CHN	0.08	0.19
ASIA	FJI	0.16	0.28
ASIA	KGZ	0.23	0.08
ASIA	IND	0.11	0.15
ASIA	IDN	0.70	0.06
ASIA	THA	0.27	0.09
ASIA	KHM	0.15	0.11
ASIA	MYS	0.76	0.14
ASIA	PHL	0.35	0.06
ASIA	VNM	0.23	0.13
ASIA	MNG	0.61	0.14
LAC	ARG	0.82	0.05
LAC	BOL	0.62	0.07
LAC	BRA	0.54	0.16
LAC	COL	0.85	0.07
LAC	CRI	0.57	0.17
LAC	CUB	0.36	0.20
LAC	DOM	0.71	0.08
LAC	ECU	0.68	0.11
LAC	SLV	0.77	0.08
LAC	HTI	0.40	0.14
LAC	JAM	0.52	0.12
LAC	MEX	0.76	0.08
LAC	NIC	0.46	0.15
LAC	PER	0.66	0.10
LAC	URY	0.84	0.04
LAC	VEN	0.12	0.13
The goodness of fit (R^2^) and error *P*-value are provided for all IPUMSI countries (including those that are not malaria endemic) listed in Table 1. Error *P*-value is here defined as the average probability that predicted migration values do not belong to the observed migration dataset.			
