# Supplementary material for: Mapping internal connectivity through human migration in malaria endemic countries
Source: Sci Data. 2016 Aug 16;3:160066. doi: 10.1038/sdata.2016.66 (PMC5127488; doi:10.1038/sdata.2016.66)
Supplement: Supplementary Tables [file sdata201666-s1.pdf]

Supplementary Figures

Table of Contents

Supplementary Table 1…….....2
Supplementary Table 2………..........5
Supplementary Table 3………..........6
Supplementary Table 4a……….........7
Supplementary Table 4b……….8
Supplementary Table 4c………9

Continent	ISO country code	Country name	Population count raster dataset	Malaria endemic for:	
				Plasmodium falciparum	***Plasmodium vivax***
Africa	AGO	Angola	WP	Y	
Africa	BDI	Burundi	WP	Y	
Africa	BEN	Benin	WP	Y	
Africa	BFA	Burkina Faso	WP	Y	
Africa	BWA	Botswana	WP	Y	
Africa	CAF	Central African Republic	WP	Y	
Africa	CIV	Côte d'Ivoire	WP	Y	
Africa	CMR	Cameroon	WP	Y	
Africa	COD	Congo (Democratic Republic of the)	WP	Y	Y
Africa	COG	Congo	WP	Y	
Africa	COM	Comoros	GPWv4		Y
Africa	DJI	Djibouti	WP	Y	Y
Africa	ERI	Eritrea	WP	Y	Y
Africa	ETH	Ethiopia	WP	Y	Y
Africa	GAB	Gabon	WP	Y	
Africa	GHA	Ghana	WP	Y	
Africa	GIN	Guinea	WP	Y	
Africa	GMB	Gambia	WP	Y	
Africa	GNB	Guinea-Bissau	WP	Y	
Africa	GNQ	Equatorial Guinea	WP	Y	
Africa	KEN	Kenya	WP	Y	Y
Africa	LBR	Liberia	WP	Y	
Africa	MDG	Madagascar	WP	Y	Y
Africa	MLI	Mali	WP	Y	
Africa	MOZ	Mozambique	WP	Y	
Africa	MRT	Mauritania	WP	Y	
Africa	MWI	Malawi	WP	Y	
Africa	MYT	Mayotte	GPWv4	Y	
Africa	NAM	Namibia	WP	Y	
Africa	NER	Niger	WP	Y	
Africa	NGA	Nigeria	WP	Y	
Africa	RWA	Rwanda	WP	Y	

Africa	SDN	Sudan	WP	Y	Y
Africa	SEN	Senegal	WP	Y	
Africa	SLE	Sierra Leone	WP	Y	
Africa	SOM	Somalia	WP	Y	Y
Africa	SSD	South Sudan	WP	Y	Y
Africa	STP	São Tomé and Príncipe	GPWv4	Y	
Africa	SWZ	Swaziland	WP	Y	
Africa	TCD	Chad	WP	Y	
Africa	TGO	Togo	WP	Y	
Africa	TZA	Tanzania, United Republic of	WP	Y	
Africa	UGA	Uganda	WP	Y	Y
Africa	ZAF	South Africa	WP	Y	
Africa	ZMB	Zambia	WP	Y	
Africa	ZWE	Zimbabwe	WP	Y	
Asia	AFG	Afghanistan	WP	Y	Y
Asia	AZE	Azerbaijan	GPWv4		Y
Asia	BGD	Bangladesh	WP	Y	Y
Asia	BTN	Bhutan	WP	Y	Y
Asia	CHN	China	GPWv4	Y	Y
Asia	GEO	Georgia	WP		Y
Asia	IDN	Indonesia	WP	Y	Y
Asia	IND	India	WP	Y	Y
Asia	IRN	Iran (Islamic Republic of)	GPWv4	Y	Y
Asia	KHM	Cambodia	WP	Y	Y
Asia	KOR	Korea (Republic of)	WP		Y
Asia	LAO	Lao People's Democratic Republic	WP	Y	Y
Asia	LKA	Sri Lanka	WP		Y
Asia	MMR	Myanmar	WP	Y	Y
Asia	MYS	Malaysia	WP	Y	Y
Asia	NPL	Nepal	WP	Y	
Asia	PAK	Pakistan	WP	Y	Y
Asia	PHL	Philippines	WP	Y	Y
Asia	PNG	Papua New Guinea	WP	Y	Y
Asia	PRK	Korea (Democratic People's Republic of)	WP		Y

Asia	SAU	Saudi Arabia	WP	Y	
Asia	SLB	Solomon Islands	WP	Y	Y
Asia	THA	Thailand	WP	Y	Y
Asia	TJK	Tajikistan	WP	Y	Y
Asia	TLS	Timor-Leste	WP	Y	Y
Asia	VNM	Vietnam	WP	Y	Y
Asia	VUT	Vanuatu	WP	Y	Y
Asia	YEM	Yemen	WP	Y	
LAC	ARG	Argentina	WP		Y
LAC	BLZ	Belize	WP		Y
LAC	BOL	Bolivia (Plurinational State of)	WP	Y	Y
LAC	BRA	Brazil	WP	Y	Y
LAC	COL	Colombia	WP	Y	Y
LAC	CRI	Costa Rica	WP		Y
LAC	DOM	Dominican Republic	WP	Y	
LAC	ECU	Ecuador	WP	Y	Y
LAC	GTM	Guatemala	WP	Y	Y
LAC	GUF	French Guiana	WP	Y	Y
LAC	GUY	Guyana	WP	Y	Y
LAC	HND	Honduras	WP	Y	Y
LAC	HTI	Haiti	WP	Y	
LAC	MEX	Mexico	WP		Y
LAC	NIC	Nicaragua	WP	Y	Y
LAC	PAN	Panama	WP	Y	Y
LAC	PER	Peru	WP	Y	Y
LAC	PRY	Paraguay	WP	Y	Y
LAC	SLV	El Salvador	WP		Y
LAC	SUR	Suriname	WP	Y	Y
LAC	VEN	Venezuela (Bolivarian Republic of)	WP	Y	Y

Supplementary Table 1. List of malaria endemic countries, according to the 2015 World Malaria Report [58] (refer to Howes et al.[63] for additional and up-to-date evidence of *Plasmodium vivax* endemicity across sub-Saharan Africa), for which 5-year (2005-2010) internal migration flows are available both through the WorldPop Project website and the WorldPop Dataverse Repository. In the 1st column, 'LAC' stands for Latin America and the Caribbean. In the 2nd column, 'WP' and 'GPWv4' indicate that total population in each administrative unit for that specific country was calculated using the corresponding WorldPop and the Gridded Population of the World version 4 dataset, respectively.

Covariate	Type	Description	Calculation
MajCeni	Binary	Whether or not the administrative unit of origin is a major unit within the corresponding country	ifelse(POPi>quantile(POPi,.9), 1,0)
MajCenj	Binary	Whether or not the administrative unit of destination is a major unit within the corresponding country	ifelse(POPj>quantile(POPj,.9), 1,0)
Tinyi	Binary	Whether or not the administrative unit of origin is a small unit within the corresponding country	ifelse(POPil<quantile(POPi,.1), 1,0))
Tinyj	Binary	Whether or not the administrative unit of destination is a small unit within the corresponding country	ifelse(POPjl<quantile(POPj,.1), 1,0))
Perci	Continuous	Percentage of population in the administrative unit of origin	POPi/sum(POPi)
Percj	Continuous	Percentage of population in the administrative unit of destination	POPj/sum(POPj)
asDensei	Continuous	ArcSin of population density of the administrative unit of origin	Asinh(UrbanPropi*100)
asDensej	Continuous	ArcSin of population density of the administrative unit of destination	Asinh(UrbanPropj*100)
asPropPopi	Continuous	ArcSin of the percentage of population in the administrative unit of origin	Asinh(Perci*100)
asPropPopj	Continuous	ArcSin of the percentage of population in the administrative unit of destination	Asinh(Percj*100)
Propij	Continuous	Relative population size of the administrative unit of origin with respect to the administrative unit of destination	POPi/POPj

Supplementary Table 2. Summary information on additional covariates (derived from the main covariates listed in Table 2) that were tested in order to improve the predictive power of the spatial gravity-type models used to estimate 5-year (2005-2010) internal human migration flows for every malaria endemic country in Supplementary Table 1.

Continent	Best predictive model	Averaged R^2
Africa	s(asDISTIJ) + URBANPROPJ + URBANPROPI + PercJ + asDENSJ + TinyI + TinyJ + CONTIJ + MajCenJ + asPOP I+ asPOPJ	0.42
Asia	s(DISTIJ)+ s(POPI) + POPJ + logAREAJ + URBANPROPI + TinyJ + MajCenJ	0.50
LAC	DISTIJ + POPI + POPJ + URBANPROPI + PercI + TinyJ + MajCenI	0.53

Supplementary Table 3. Summary information on the best prediction model identified for each continent. In the 1st column, LAC stands for Latin America and the Caribbean. In the 2nd column, *s()*, *as* and *log* indicate for which covariates linear predictors were replaced by additive predictors, which covariates were arcsine and log-transformed (after being converted to proportions), respectively. Refer to Table 2 and Supplementary Table 2 for the meaning of the covariate acronyms in the 2nd column and for a description of how these covariates were calculated.

Linear term<br>(Covariate description)	Estimated coefficient	Std.Error	z value	p-value
(Intercept)	-6.63	0.0156	-426.41	<0.0001
URBANPROPJ<br>(Proportion of urban population in the administrative unit of destination)	0.96	0.0052	184.26	<0.0001
URBANPROPI<br>(Proportion of urban population in the administrative unit of origin)	1.12	0.0048	235.38	<0.0001
PercJ<br>(Percentage of population in the administrative unit of destination)	-0.21	0.0031	-67.44	<0.0001
asDENSJ<br>(ArcSin of population density of the administrative unit of destination)	-0.02	0.0008	-28.02	<0.0001
TinyI<br>(Whether or not the administrative unit of origin is a small unit within the corresponding country)	-0.05	0.0046	-11.50	<0.0001
TinyJ<br>(Whether or not the administrative unit of destination is a small unit within the corresponding country)	0.44	0.0049	90.27	<0.0001
CONTIJ<br>(Contiguity between administrative units)	0.99	0.0025	403.60	<0.0001
MajCenJ<br>(Whether or not the administrative unit of destination is a major unit within the corresponding country)	0.64	0.0027	238.74	<0.0001
asPOPI<br>(ArcSin of the total population in the administrative unit of origin)	0.47	0.0010	449.99	<0.0001
asPOPJ<br>(ArcSin of the total population in the administrative unit of destination)	-0.53	0.0012	-440.36	<0.0001
**GAM term<br>(Covariate description)**	**EDF**	**Ref.df**	**Chi.sq**	**p-value**
s(asDISTIJ)<br>(ArcSin of the distance between the administrative unit of origin and destination)	1.96	2.00	70084	<0.0001

Supplementary Table 4a. Summary statistics for the top predictive model for Africa. 'Estimated coefficient' indicates the coefficient used in the model for each linear term, 'Std.Error' indicates the standard error of the 'Estimate coefficient', 'z-value' is the computed z-score for each coefficient used to calculate the p-value for each linear term. EDF indicates the estimated degrees of freedom for each GAM (nonlinear) term, Ref.df indicates the residual degrees of freedom, Chi.sq indicates the Chi-squared value used to calculate the p-value for each smooth nonlinear term.

**Linear term**<br>**(Covariate description)**	**Estimated coefficient**	**Std.Error**	**z value**	**p-value**
(Intercept)	-6.53	0.0069	-946.253	<0.0001
POPJ<br>(Total population in the administrative unit of destination)	0.0001	0.0001	-70.633	<0.0001
LogAREAJ<br>(Log of the surface area of the administrative unit of destination)	-0.0292	0.0006	-45.158	<0.0001
URBANPROPI<br>(Proportion of urban population in the administrative units of origin)	1.43	0.0042	338.752	<0.0001
TinyJ<br>(Whether or not the administrative unit of origin is a small unit within the corresponding country)	-0.327	0.0046	-71.919	<0.0001
MajCenJ<br>(Whether or not the administrative unit of destination is a major unit within the corresponding country)	0.19	0.0023	81.735	<0.0001
**GAM term**<br>**(Covariate description)**	**EDF**	**Ref.df**	**Chi.sq**	**p-value**
s(DISTIJ)<br>(Distance between the administrative unit of origin and destination)	2.00	2.00	434069.32	<0.0001
s(POPI)<br>(Total population in the administrative unit of origin)	2.00	2.00	224632.90	<0.0001

Supplementary Table 4b. Summary statistics for the top predictive model for Asia. 'Estimated coefficient' indicates the coefficient used in the model for each linear term, 'Std.Error' indicates the standard error of the 'Estimate coefficient', 'z-value' is the computed z-score for each coefficient used to calculate the p-value for each linear term. EDF indicates the estimated degrees of freedom for each GAM (nonlinear) term, Ref.df indicates the residual degrees of freedom, Chi.sq indicates the Chi-squared value used to calculate the p-value for each smooth nonlinear term.

Linear term<br>(Covariate description)	Estimated coefficient	Std.Error	z value	p-value
(Intercept)	-5.17	0.013	-396.14	<0.0001
AsDISTIJ<br>(ArcSin of the distance between the administrative unit of origin and destination)	-0.73	0.001	-780.84	<0.0001
AsPOPI<br>(ArcSin of the total population in the administrative unit of origin)	0.37	0.001	356.63	<0.0001
AsPOPJ<br>(ArcSin of the total population in the administrative unit of destination)	-0.16	0.001	-175.51	<0.0001
URBANPROPI<br>(Proportion of urban population in the administrative unit of origin)	0.96	0.005	213.95	<0.0001
PercI<br>(Percentage of population in the administrative unit of origin)	-0.16	0.003	-48.17	<0.0001
TinyJ<br>(Whether or not the administrative unit of destination is a small unit within the corresponding country)	0.07	0.005	15.17	<0.0001
MajCenI<br>(Whether or not the administrative unit of origin is a major unit within the corresponding country)	0.33	0.003	118.29	<0.0001

Supplementary Table 4c. Summary statistics for the top predictive model for Latin America and the Caribbean. 'Estimated coefficient' indicates the coefficient used in the model for each linear term, 'Std.Error' indicates the standard error of the 'Estimate coefficient', 'z-value' is the computed z-score for each coefficient used to calculate the p-value for each linear term.
