# Supplementary material for: Mapping internal connectivity through human migration in malaria endemic countries
Source: Sci Data. 2016 Aug 16;3:160066. doi: 10.1038/sdata.2016.66 (PMC5127488; doi:10.1038/sdata.2016.66)
Supplement: Supplementary Figures [file sdata201666-s2.pdf]

Supplementary Figures

Table of Contents

Supplementary Figure 1……………………………………………………………………………………………………2
Supplementary Figure 2a……………………………………………………………………………………………………3
Supplementary Figure 2b……………………………………………………………………………………………………4
Supplementary Figure 3……………………………………………………………………………………………………5

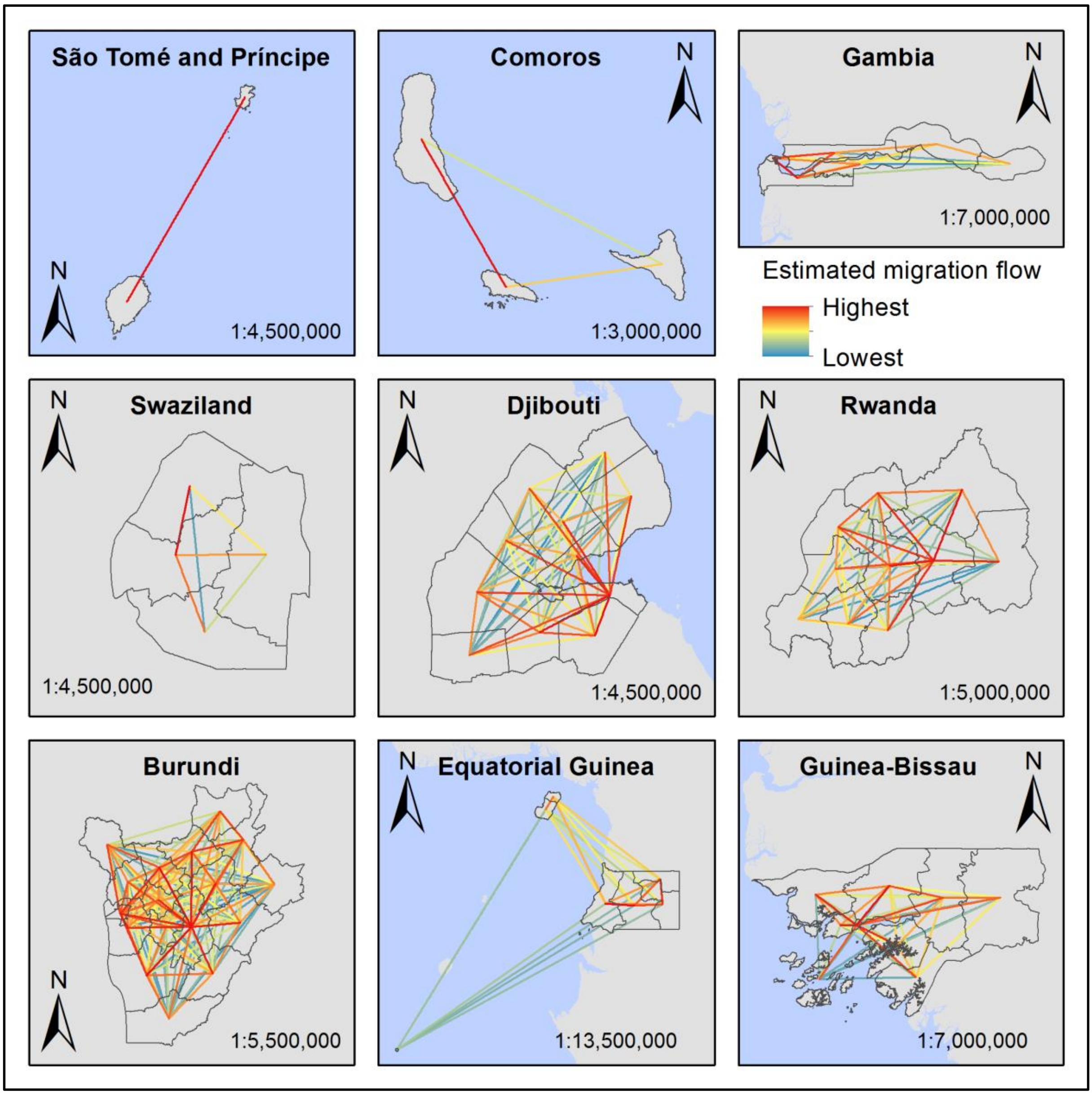

Supplementary Figure 1. *Close-up views of internal migration flows in São Tomé and Príncipe, Comoros, Gambia, Swaziland, Djibouti, Rwanda, Burundi, Equatorial Guinea, and Guinea-Bissau.*

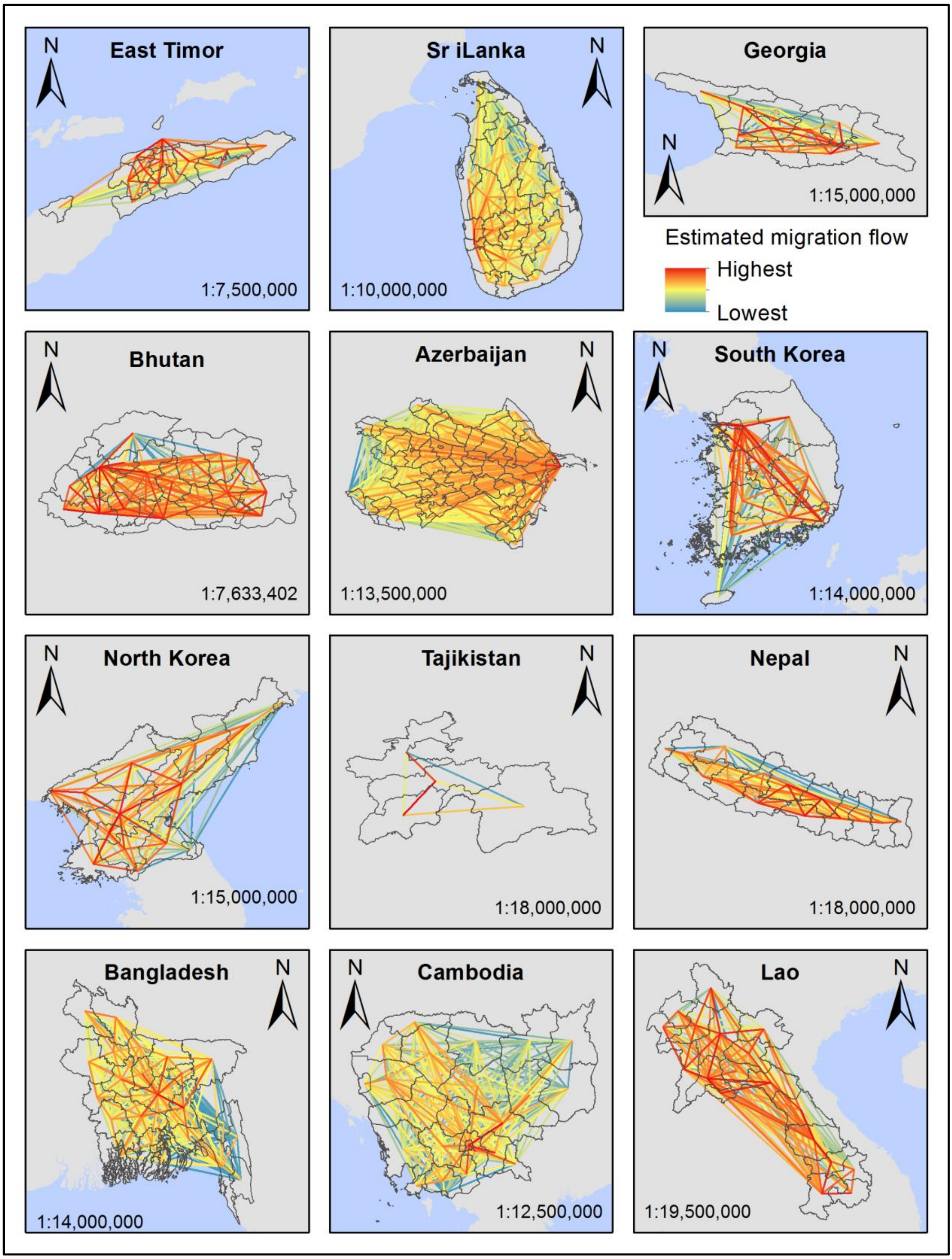

Supplementary Figure 2a. *Close-up views of internal migration flows in East Timor, Sri Lanka, Georgia Bhutan, Azerbaijan, South and North Korea, Tajikistan, Nepal, Bangladesh, Cambodia, and Lao.*

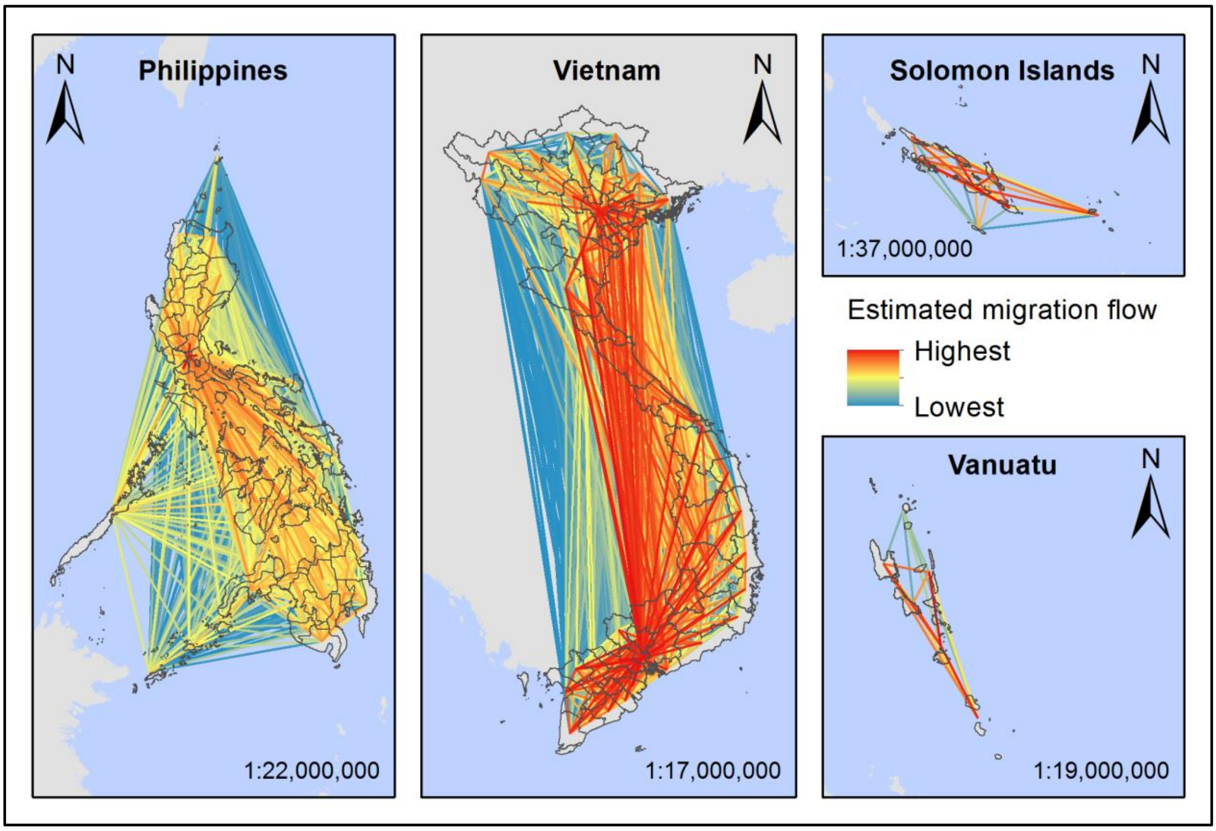

Supplementary Figure 2b. Close-up views of internal migration flows in Philippines, Vietnam, Salomon Islands, and Vanuatu.

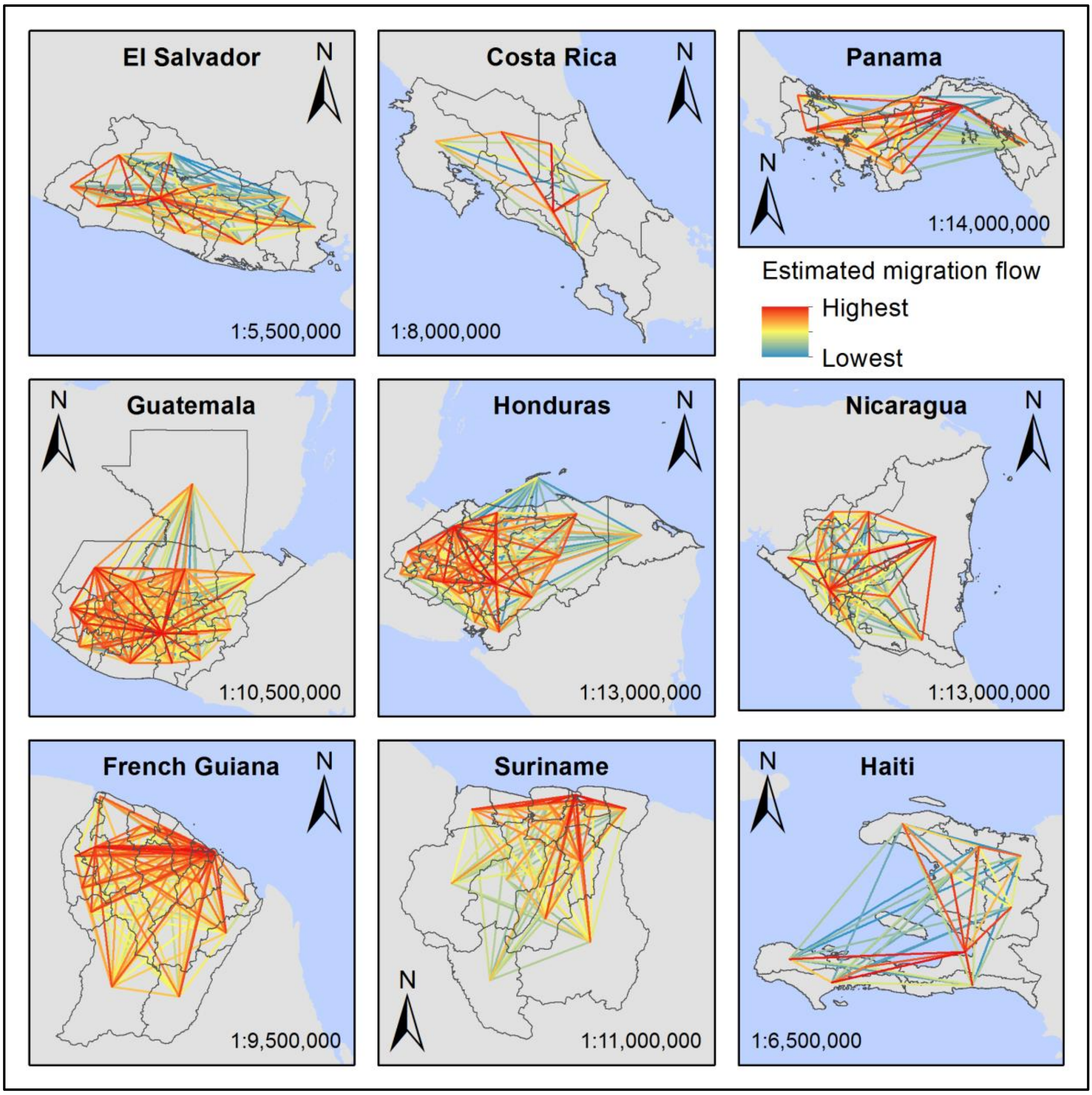

Supplementary Figure 3. *Close-up views of internal migration flows in El Salvador, Costa Rica, Panama, Guatemala, Honduras, Nicaragua, French Guiana, Suriname, and Haiti.*
